# A Successful Case of Complete Resection With Preservation of the Right Coronary Artery in an Infiltrative Cardiac Hemangioma Using Indocyanine Green Fluorescence Angiography

**DOI:** 10.7759/cureus.92243

**Published:** 2025-09-13

**Authors:** Ren Saito, Yujiro Miura, Atsuyuki Mitsuishi, Miho Tsutsui, Tatsuya Noguchi

**Affiliations:** 1 Cardiovascular Surgery, Kochi Medical School Hospital, Nankoku, JPN; 2 Cardiothoracic Surgery, Kochi Medical School Hospital, Nankoku, JPN; 3 Pathology, Kochi Medical School Hospital, Nankoku, JPN; 4 Cardiology and Geriatrics, Kochi Medical School Hospital, Nankoku, JPN

**Keywords:** bypass, cardiac hemangioma, cardiac tumors, echocardiography, icg fluorescence angiography, indocyanine green

## Abstract

Cardiac hemangiomas are rare benign tumors that can arise anywhere in the heart. Complete surgical resection is recommended; however, if the tumor is extensive and invades the surrounding structures, resection may require sacrificing involved tissue. This report describes a case in which a substantial cardiac hemangioma encircling the right coronary artery was completely excised using epicardial echocardiography and indocyanine green fluorescence angiography. We discuss the utility of these modalities during surgery.

## Introduction

Primary cardiac tumors are rare, with an autopsy prevalence of 0.03%. Cardiac hemangiomas account for only 1-2% of these tumors [1]. They can occur anywhere in the heart [2], with the most common site being the right ventricle, followed by the left ventricle and right atrium [3]. Cases involving the main trunk of the right coronary artery are rare, and resection may require vascular reconstruction or coronary artery bypass grafting [4,5]. In such cases, both complete resection of the tumor and preservation of the coronary artery are key surgical considerations. In the present case, the tumor extended from the right atrium to the right ventricle and encircled the right coronary artery. Such tumors often exhibit slow growth and may remain asymptomatic, leading to delayed detection of large masses that sometimes involve multiple chambers of the heart [4]​​​​.

## Case presentation

**Figure 1:**
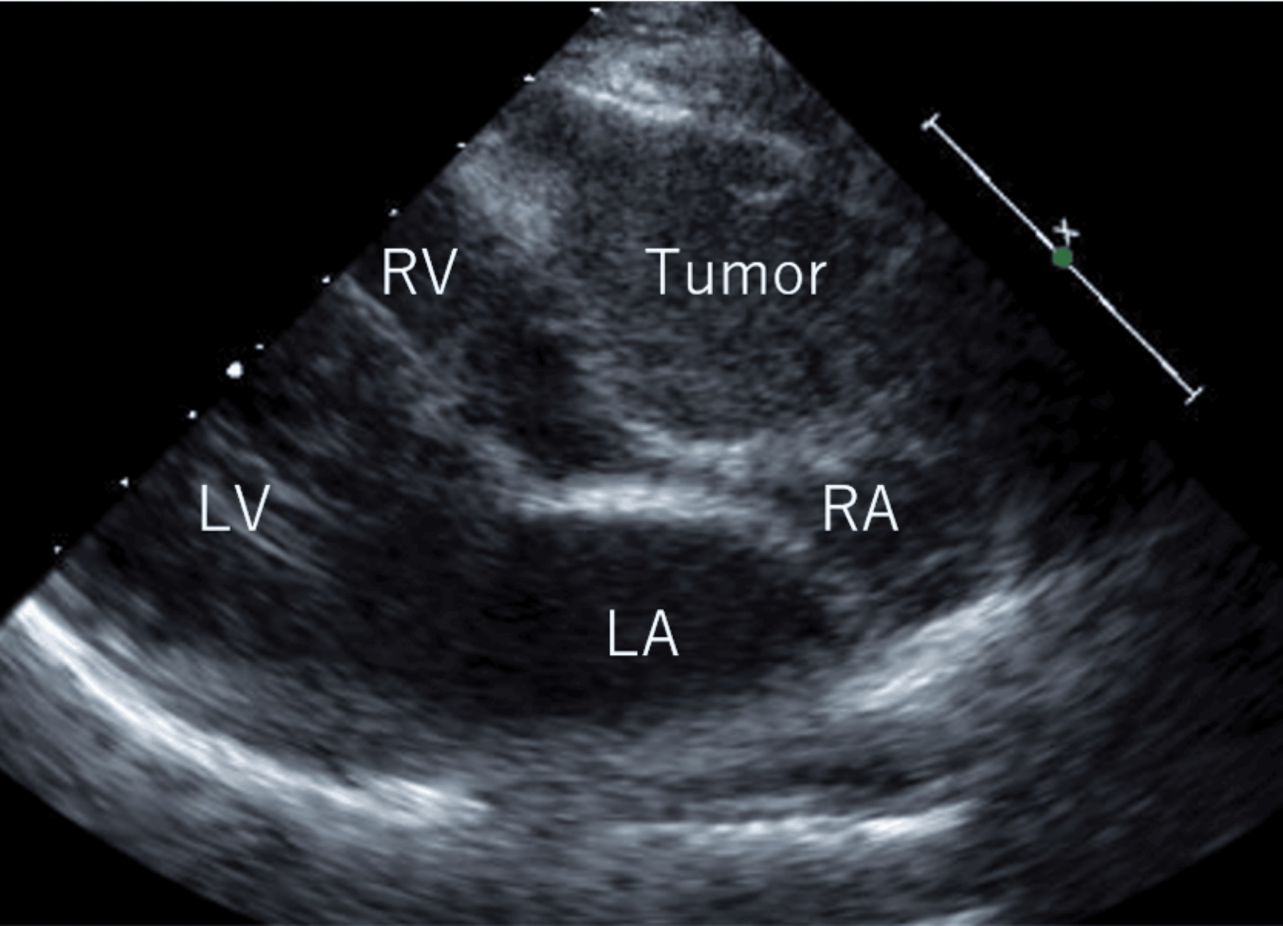
Preoperative echocardiography. Echocardiography revealed abnormal structures in the right atrium. RA: right atrium; LA: left atrium; LV: left ventricle; RV; right ventricle

A 55-year-old woman underwent a preoperative examination for an ovarian tumor, during which echocardiography revealed an abnormal structure in the right atrium (Figure 1).

**Figure 2:**
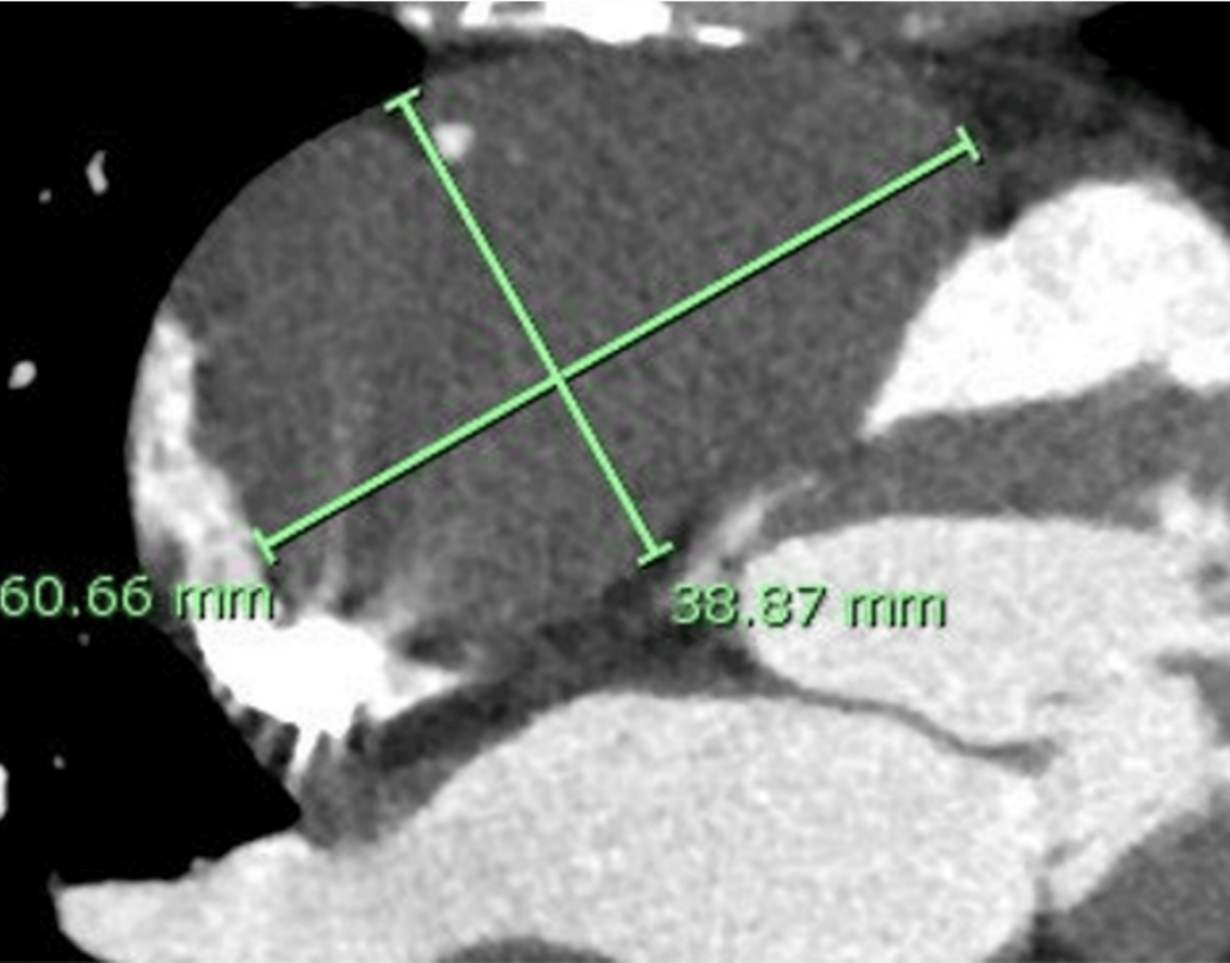
Preoperative computed tomography. Computed tomography imaging showed a low-attenuation mass on the anterior surface of the right atrium.

She was referred to the Department of Cardiology. Imaging studies suggested a cardiac hemangioma, and she was kept under observation. Over a 30-month follow-up period, progressive enlargement was noted, raising concerns about potential tricuspid valve involvement, which prompted referral to the surgical department. Laboratory test results, including tumor markers, were normal. A 12-lead electrocardiogram showed sinus rhythm without notable ST-T changes. Transthoracic echocardiography revealed a left ventricular diastolic/systolic diameter of 38/20 mm, an ejection fraction of 65%, and mild-to-moderate tricuspid regurgitation. An epicardial mass was observed along the anterior surface of the ascending aorta, extending to the right atrium, mid-right ventricle, and anterior wall of the right ventricular outflow tract. Contrast-enhanced computed tomography demonstrated a low-attenuation mass on the anterior surface of the right atrium (Figure 2).

**Figure 3:**
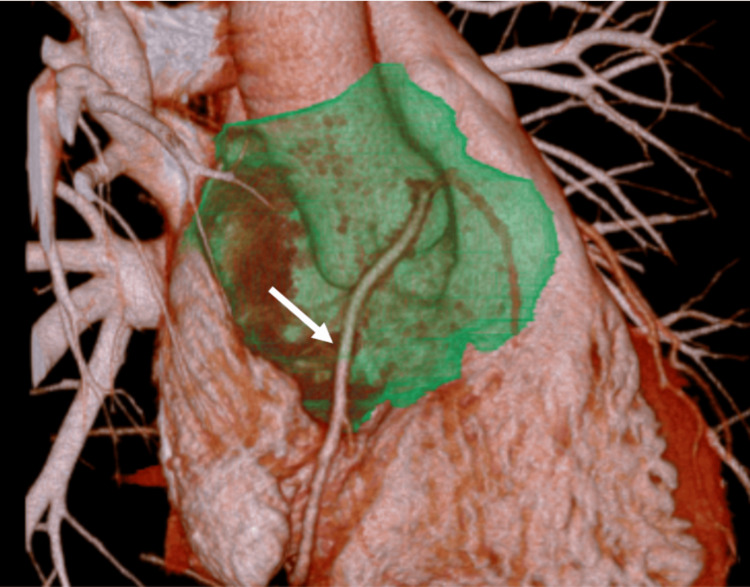
Preoperative computed tomography. The main trunk of the right coronary artery runs across the tumor (arrowhead).

The main trunk of the right coronary artery (RCA) traversed the tumor (Figure 3).

**Figure 4:**
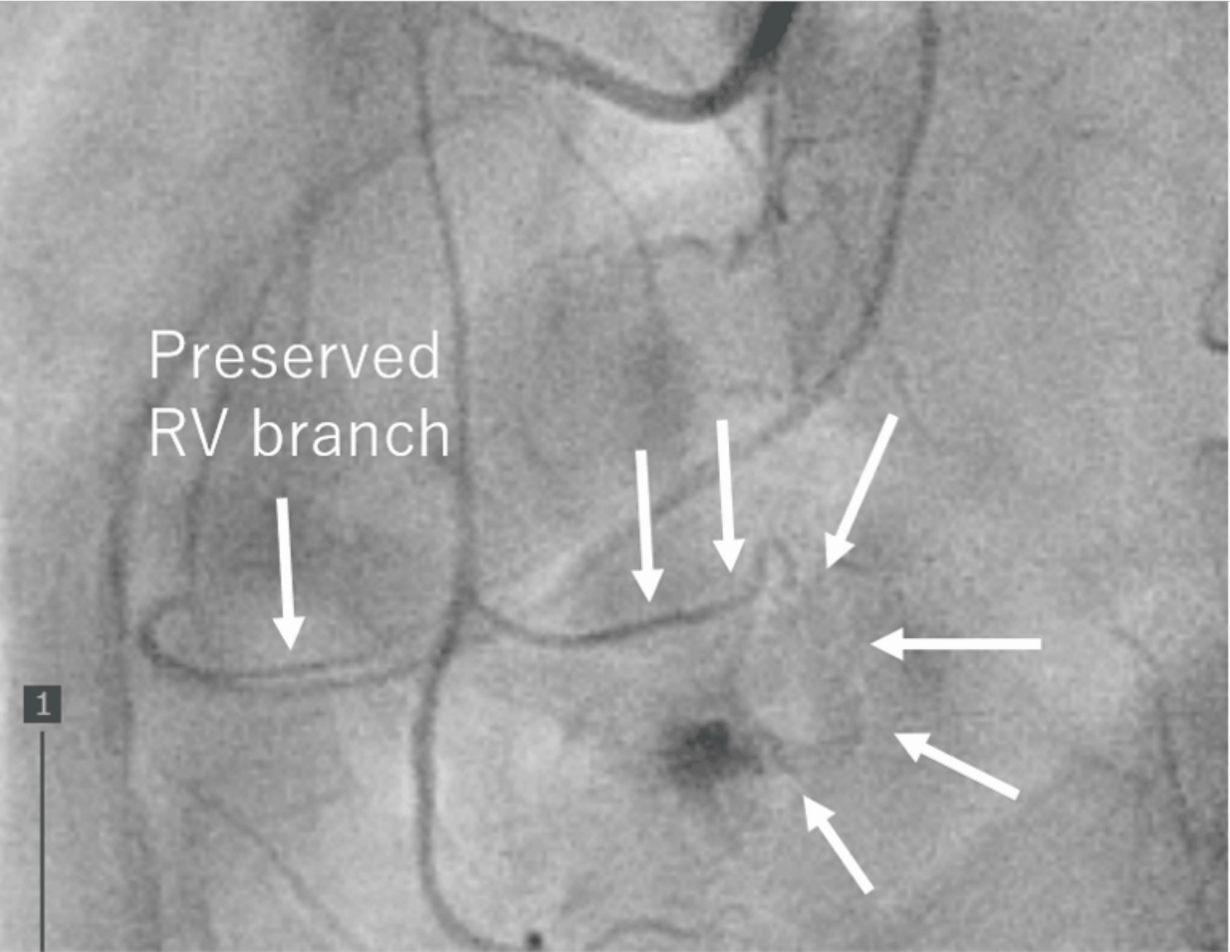
Preoperative coronary angiography. Coronary angiography revealed a feeding branch from the right coronary artery to the hemangioma (arrowhead). RV: right ventricle

The lesion had well-defined borders, an elevated apparent diffusion coefficient, and markedly high signal intensity on T2-weighted contrast-enhanced magnetic resonance imaging. The tumor exhibited progressive contrast enhancement from the periphery. Coronary angiography confirmed a feeding branch from the RCA to the hemangioma (Figure 4).

**Figure 5:**
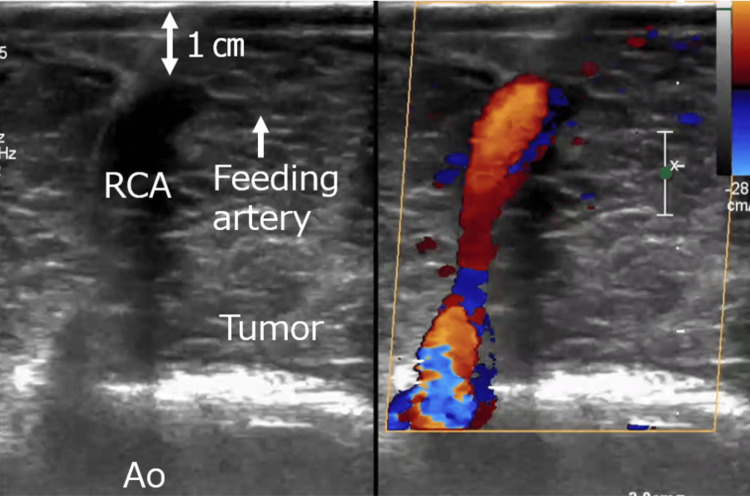
Intraoperative periepicardial echocardiography. The course of the coronary artery within the tumor was visualized using periepicardial echocardiography. RCA: right coronary artery

**Figure 6:**
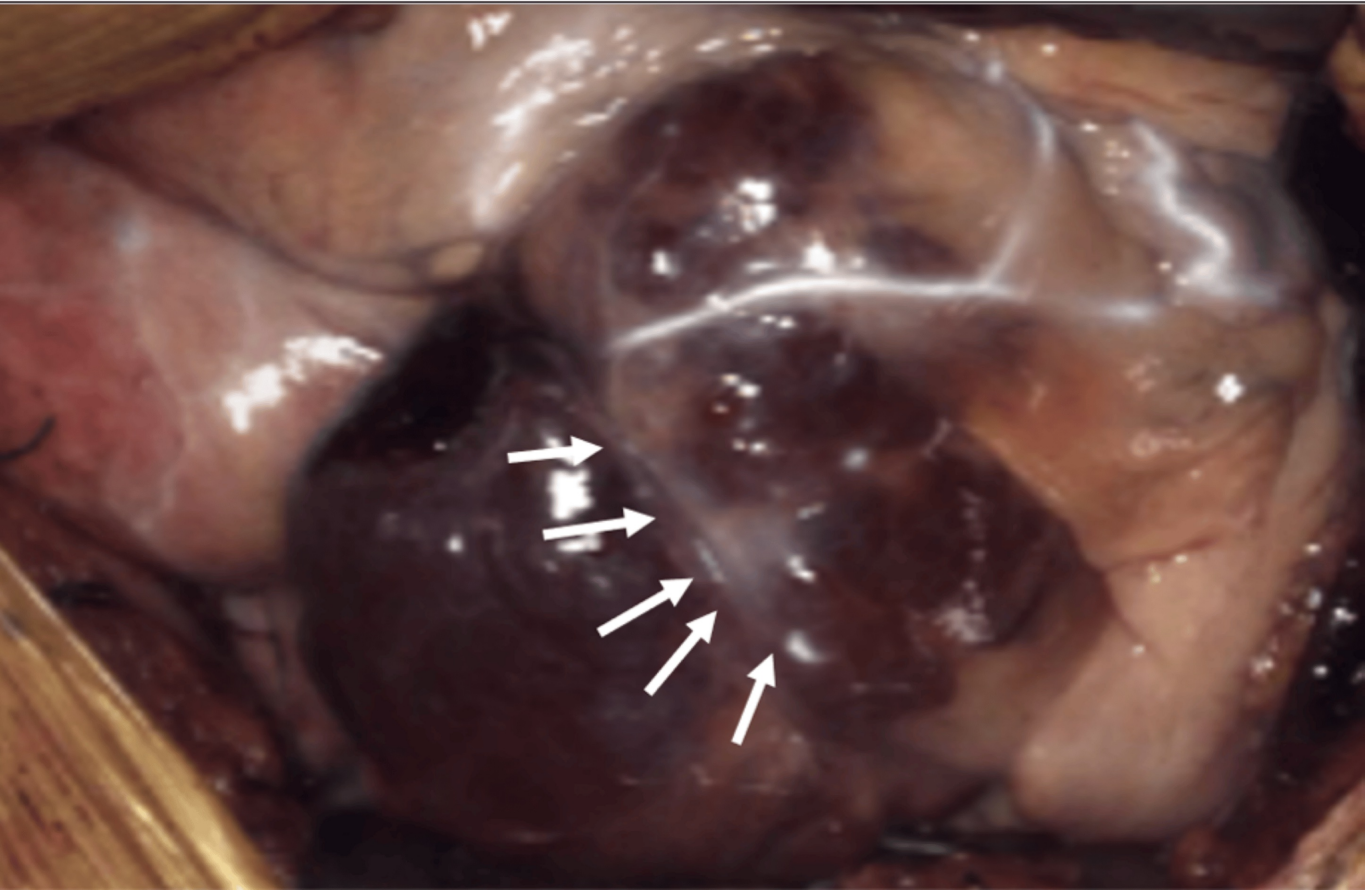
Intraoperative indocyanine green (ICG) imaging. Branches originating from the right coronary artery (RCA) within the cardiac tumor were identified and marked using indocyanine green (ICG) imaging (arrowhead).

Based on these imaging findings, a cardiac hemangioma was considered the most probable diagnosis. The surgical plan assumed that the tumor had infiltrated or encircled the RCA. Surgery commenced with a median sternotomy, and the saphenous vein graft (SVG) was procured from the right lower leg for revascularization. The tumor was located between the right atrium and right ventricle and exhibited a reddish-brown hue. Intraoperative periepicardial echocardiography and indocyanine green (ICG) fluorescence angiography were used to outline the RCA and its tumor-feeding branches (Figures 5, 6).

**Figure 7:**
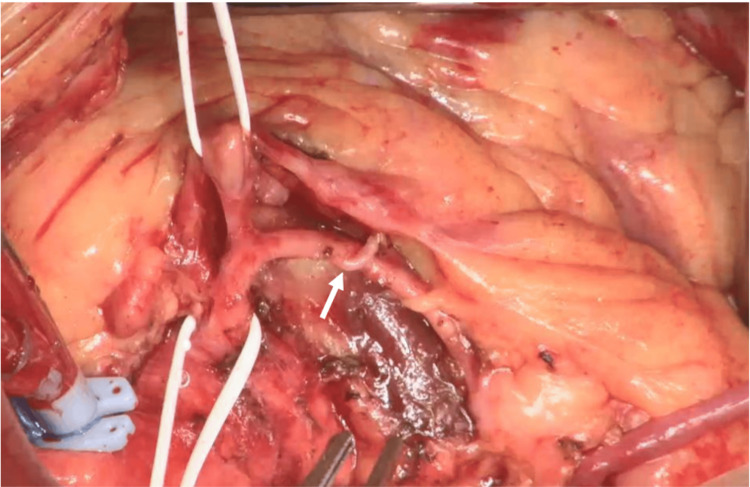
Intraoperative photograph. The tumor was resected by dividing only the feeding branches arising from the right coronary artery without injuring the main trunk (arrowhead).

The demarcation line between the tumor and vessel was distinct, indicating that preservation of the RCA was feasible. After heparin administration, cardiopulmonary bypass was established with arterial perfusion via the ascending aorta and venous drainage via the inferior vena cava. The intraoperative pathological diagnosis was consistent with cardiac hemangioma. The ascending aorta was clamped, and a cardioplegic solution was administered anterogradely to achieve cardiac arrest. The midsection of the RCA was secured to prevent injury, and the SVG was anastomosed. The tumor was carefully dissected from the RCA using a HARMONIC FOCUS™ Shears (Ethicon Inc., Raritan, New Jersey, United States). The boundary between the right atrium, right ventricle, and tumor was clearly identified, allowing mobilization of the tumor and preservation of the entire RCA without injury. A feeding arteriole near the right ventricular branch was sacrificed to remove the tumor along with its capsule (Figure 7).

**Figure 8:**
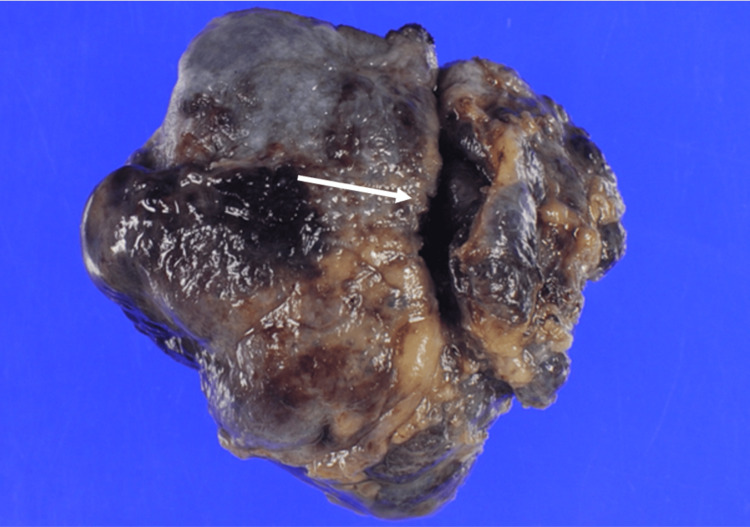
Histopathologic slide. The preserved portion of the right coronary artery (RCA) appeared fissure-like.

After restoring the heartbeat, transesophageal echocardiography revealed no abnormalities in the right heart chambers, and RCA flow was normal (>100 mL/min; pulsatility Index, 1.0). The SVG was sacrificed. The patient was weaned off cardiopulmonary bypass with minimal inotropic support. The durations of myocardial ischemia and cardiopulmonary bypass were 69 and 106 minutes, respectively. On pathological examination, the tumor had a smooth surface, and the area where the RCA was preserved appeared fissure-like (Figure 8).

**Figure 9:**
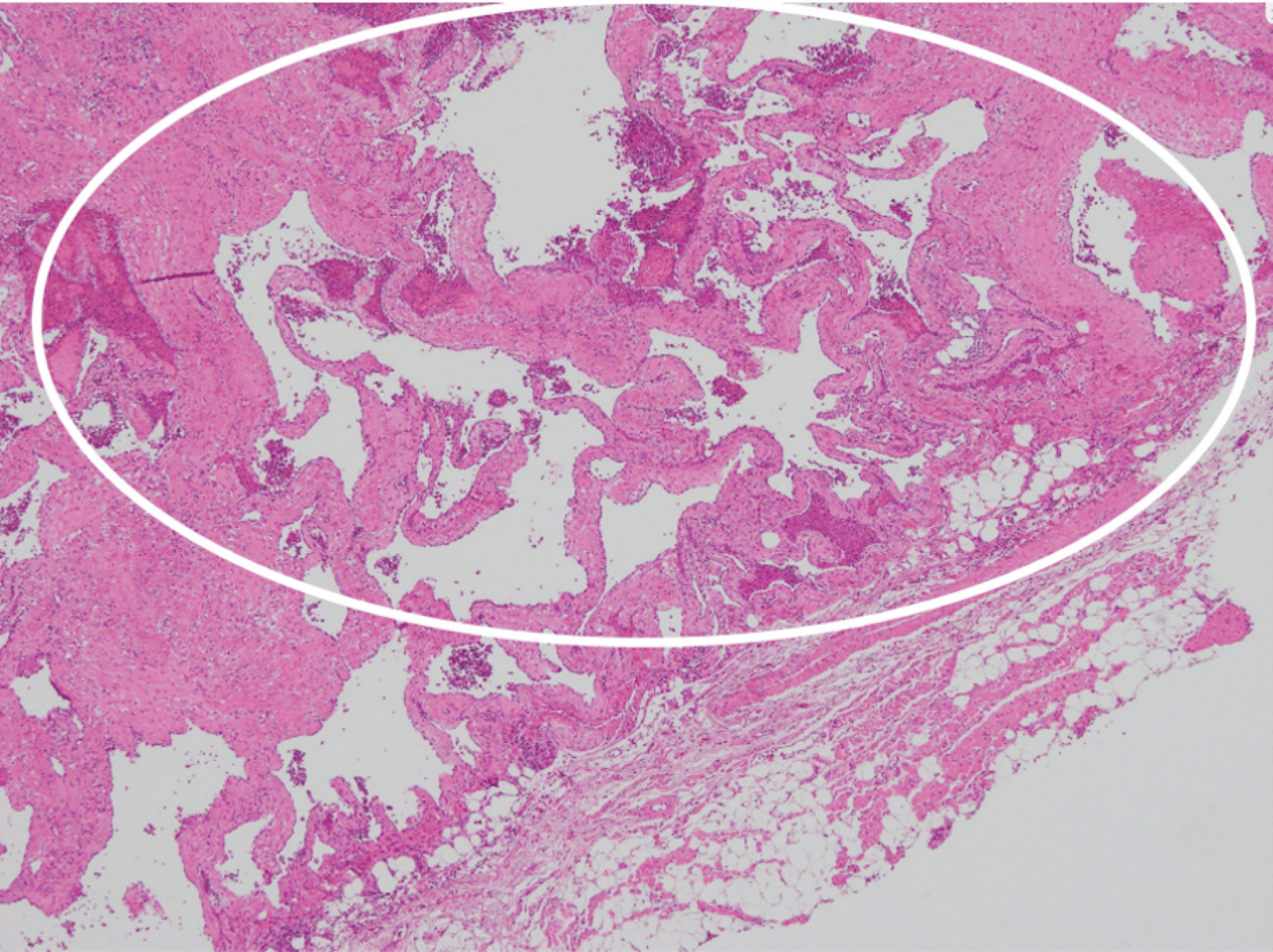
Histopathologic slide. Hematoxylin and eosin staining at 40 × magnification showed enlarged, irregular blood vessels forming anastomoses and densely distributed in the circled area. A venous malformation, specifically, a cavernous hemangioma, was diagnosed.

Histological analysis revealed densely distributed, enlarged, irregular blood vessels forming anastomoses within a relatively well-defined lesion. A venous malformation, specifically, a cavernous hemangioma, was diagnosed. The primary site of the lesion was the epicardial adipose tissue, with no obvious exposure of the cut ends (Figure 9).

The patient recovered uneventfully and remained in good clinical condition under periodic monitoring, with no evidence of recurrence.

## Discussion

This case involved a cardiac hemangioma encasing the RCA, in which echocardiography and ICG fluorescence angiography were used to confirm the vascular anatomy, facilitating complete resection without arterial injury. A similar report described accidental injury to the RCA [5], which required coronary artery bypass grafting. To prevent such complications, a prophylactic bypass was prepared by anastomosing the great saphenous vein to the distal RCA beyond the tumor. Ultimately, the tumor was resected without arterial damage. In retrospect, assessing the need for bypass based on the ease of separation between the tumor and vessel might have been a viable approach.

Cardiac hemangiomas are generally well demarcated from surrounding tissues, facilitating surgical dissection. However, when adjacent to critical structures such as coronary arteries or the myocardium, careful dissection is essential. While epicardial echocardiography is convenient, noninvasive, and useful for intraoperative vessel localization [6], its utility may be limited when the coronary artery is obscured by tumor tissue. The combination of epicardial echocardiography and ICG fluorescence angiography provided complementary visual information, enabling precise identification of the RCA and safe tumor resection along the capsule without injury.

ICG fluorescence angiography allows real-time visualization of blood flow to measure perfusion, lumen size, and distribution, providing angiography-like results without requiring radiography [7]. Using a dedicated camera system, intravenous ICG can be imaged without nephrotoxic contrast agents or ionizing radiation. ICG fluorescence angiography is a safe and reproducible technique for intraoperative vascular assessment, particularly in cardiovascular surgery [8]. One drawback is its limited tissue penetration. The combination of ICG fluorescence angiography and echocardiography facilitated the identification of the RCA and its right ventricular branches. This approach ensured RCA preservation during complete tumor resection by addressing only the feeding branches, thereby demonstrating its efficacy. Regardless of hemangioma subtypes, incomplete resection or unresectable cases are associated with poor prognosis. Therefore, complete resection encompassing the tumor attachment points is essential. Some reports indicate recurrence as late as seven or 10 years postoperatively, highlighting the need for meticulous long-term follow-up [9,10]​​​​​.

## Conclusions

In this case, the main trunk of the RCA traversed the tumor. The combined use of epicardial echocardiography and ICG fluorescence angiography enhances intraoperative visualization, enabling safe and thorough resection of cardiac hemangiomas, even when adjacent to critical structures such as the RCA.
